# Supplementary material for: Results of a fast-track HIV and hepatitis C screening protocol in Barcelona, Spain
Source: PLoS One. 2025 Oct 14;20(10):e0328464. doi: 10.1371/journal.pone.0328464 (PMC12520359; doi:10.1371/journal.pone.0328464)
Supplement: Attachment — Submitted filename: 22130 20250626 Response to Reviewers.docx [file pone.0328464.s001.docx]

**Manuscript PONE-D-24-57558**

***“Results of a fast-track HIV and hepatitis C screening protocol in Barcelona, Spain”***

**Dear Dr. Khudyakov,**

**We would like to thank you and the reviewer for your thorough evaluation of our manuscript and for the constructive feedback provided. We appreciate the opportunity to revise our work and have taken great care to address all the points raised.**

**In response to the reviewer’s comments, we have implemented substantial revisions to improve the clarity, structure, and methodological transparency of the manuscript. This includes:**

- **A clearer presentation of the study rationale and objectives**
- **Expanded descriptions of the FOCUS TEST model and the screening protocol**
- **Specification of the immunoassays and laboratory methods used**
- **Inclusion of additional statistical details and planned comparative analyses**
- **A more contextualized Introduction, Discussion and Conclusion sections**

**We have also revised the language throughout the manuscript to ensure consistency, clarity, and a uniform third-person narrative. A detailed, point-by-point rebuttal to the reviewer’s comments is placed below.**

**We hope that these revisions fully address the concerns raised and that the manuscript is now suitable for publication in *PLOS ONE*. We remain at your disposal for any additional clarifications that may be required.**

**Thank you again for your time and consideration.**

**Sincerely,**
Dr. Iván Chivite Ferriz (on behalf of all co-authors)
Hospital Clínic de Barcelona
ichivite@clinic.cat

**Point-by-point response**

***********************************************************

**Review Comments to the Author**

**Reviewer #1: Results of a fast-track HIV and hepatitis C screening protocol in Barcelona, Spain**

**1. Summary of the Research**

**The study assessed the outcomes of a fast-track HIV and hepatitis C (HCV) screening protocol implemented in the emergency department of Hospital Clínic de Barcelona. The authors report that they conducted a retrospective observational study, with subjects being adults presenting with genitourinary complaints or high-risk exposures. They used the FOCUS TEST model and achieved a testing uptake of 70% for HIV and 92% for HCV, identifying 38 new HIV infections and 34 new HCV infections. Linkage to care was successfully achieved for 89% of new HIV cases and 100% of new HCV cases. The study highlights the feasibility and effectiveness of systematic opportunistic screening in emergency settings, emphasizing the need for dedicated personnel to ensure high linkage to care rates.**

**The manuscript has a number of issues that need to be addressed. I recommend that the manuscript needs major revisions.**

**2. Examples and Evidence**

**2.1. Major Issues**

**2.1.1. The Abstract**

**• Lack of Specificity on the model: The abstract mentions use of the FOCUS TEST model but does not provide information on the model. It is suggested that the authors include a very brief description of the FOCUS TEST model to help readers understand the framework and its application.**

**Response:**
We thank the reviewer for this valuable suggestion. We agree that briefly describing the FOCUS TEST model in the Abstract would enhance clarity and help readers unfamiliar with the model to better understand the framework used in our study. We have therefore added a concise description of the model in the revised Abstract.

**Revised Abstract text (addition in Italic):**
"Using the FOCUS TEST model as a framework, we conducted systematic opportunistic HIV and HCV screening and offered linkage to care (LTC) to patients with positive test results. *The FOCUS TEST model supports automatic integration of testing into routine workflows and staff training.*"

**• No description of the project in the abstract: The abstract refers to the project but does not clearly indicate what the project is. The authors could include a very short description of the project in the abstract to help readers understand the context and objectives of the study more clearly.**

**Response:**
We thank the reviewer for this helpful observation. To improve clarity and provide better context, we have included a brief description of the project in the Abstract. This addition outlines the scope and purpose of the screening initiative more explicitly.

**Revised Abstract text (addition in *italic*):**
"This study assessed the outcomes of a *healthcare quality improvement project integrating routine opportunistic BBV screening and linkage to care in emergency services for high-risk patients. This* project aimed at providing HIV and HCV fast-track screening among patients seeking care in the emergency department of Hospital Clínic de Barcelona (Spain)."

**• Patients re-linked to care are not adequately addressed in the abstract: The separate set of patients re-linked to care are only mentioned in the results section of the abstract, which limits the reader's understanding of the full scope and impact of the study. It is proposed that the authors briefly mention this group in the abstract introduction to set the context, in the abstract methods section to explain the process of re-linking these patients to care, and in the abstract conclusion to highlight re-engaging previously diagnosed patients.**

**Response:**
We thank the reviewer for this thoughtful and constructive suggestion. We agree that explicitly referencing the re-linkage to care of previously diagnosed patients throughout the Abstract would better reflect the full scope and public health relevance of the project. We have revised the Abstract accordingly, introducing this group of patients in the introduction and highlighting the impact of this component in the conclusion. However, the linkage to care (LTC) process was already described in methods

**Revised Abstract text (additions in *italic*):**

- **Introduction:**
  "project aimed at providing HIV and HCV fast-track screening among patients seeking care in the emergency department of Hospital Clínic de Barcelona (Spain) *and re-engage individuals previously diagnosed but not currently in care.*"
- **Conclusion:**
  "This healthcare quality improvement project was feasible and successful in achieving its goal of providing systematic opportunistic HIV and HCV screening to patients seeking urgent care. *Importantly, the program also enabled LTC of a considerable number of patients previously diagnosed but not retained in care, further strengthening its impact on public health.*"

**• Conclusion too brief and non-specific: The conclusion does not explicitly connect the project's success to broader public health goals and its potential for scalability or application in other settings is not addressed. It is suggested that the authors link the findings to HIV and HCV elimination goals and include the potential for broader application of the findings.**

**Response:**
We appreciate the reviewer’s insightful comment. We agree that strengthening the conclusion of the abstract by linking our results to global public health goals and potential applicability in other settings would enhance its relevance and impact. Accordingly, we have revised the Abstract’s conclusion to explicitly mention the WHO HIV/HCV elimination targets and the program’s potential for broader implementation.

**Revised Abstract text (addition in *italic*):**
"This healthcare quality improvement project was feasible and successful in achieving its goal of providing systematic opportunistic HIV and HCV screening to patients seeking urgent care. Importantly, the program also enabled LTC of a considerable number of patients previously diagnosed but not retained in care, further strengthening its impact on public health. *These outcomes align with global goals for the elimination of HIV and HCV as public health threats by 2030 and demonstrate that similar fast-track screening and linkage strategies could be effectively implemented in other urban emergency settings with appropriate infrastructure and support.*"

**2.1.2. Introduction**

**• Specificity of Previous Studies: General statistics and the importance of screening are mentioned, but the study does not describe in more detail specific previous studies that have addressed similar issues. Highlighting specific gaps or limitations in past research could better justify the need for this study.**

**Response:**
We thank the reviewer for this pertinent suggestion. We agree that including more specific references to previous studies and explicitly identifying gaps in the literature would strengthen the rationale for our study. We have revised the Introduction accordingly.

**Revised Introduction text (addition in *italic*):**
"*Although the importance of HIV and HCV screening in EDs is well recognized, few studies have implemented integrated strategies combining systematic screening, automation, coinfection assessment, and re-linkage to care. For example, the systematic review by Simmons et al. (9) reported a wide range of HIV and HCV seroprevalence rates in ED settings (up to 13% for HIV in certain contexts), as well as significant variability in acceptability and implementation. One of the key findings was that, although screening was feasible, LTC rates were often suboptimal and inconsistently reported across centers.*

*Other studies, such as Hoxhaj et al. (13), evaluated opt-out HIV screening using non-rapid technology in high-volume EDs but did not include HCV or report LTC outcomes. Freeman et al. (12) documented good patient acceptance of rapid HIV testing in a southeastern ED but did not address automation or continuity of care. More recently, Vaz-Pinto et al. (14) demonstrated that automated algorithms integrated into the electronic medical record improved early HIV diagnosis in Portuguese EDs; however, their study focused solely on HIV, did not include HCV, and did not report data on re-linkage of previously diagnosed patients. In this context, there remains a clear need for studies that go beyond assessing feasibility and uptake of HIV and HCV screening, and that report comprehensive outcomes including seroprevalence, coinfection rates, LTC, and effective strategies for re-engaging patients lost to follow-up.*”

**• Detailed Discussion on Linkage to care (LTC) Challenges: Although the introduction mentions that LTC remains sub-optimal, it could benefit from a more detailed discussion on why LTC is challenging and what specific barriers exist, which would provide a clearer context for the study's focus on improving LTC rates.**

**Response:**
We appreciate the reviewer’s insightful comment. We agree that expanding the discussion in the Introduction to outline the known barriers to successful linkage to care (LTC) would provide a clearer justification for the design of our intervention. We have revised the text accordingly to include these challenges.

**Revised Introduction text (addition in *italic*):**
"Although linkage to care (LTC) upon BBV screening in EDs is still sub-optimal, screening in this setting could be used to re-engage patients who are already aware of their infection but not yet linked to care [14–16]. *Challenges to effective LTC include lack of patient awareness of the importance of follow-up, structural barriers such as transportation or clinic accessibility, stigma associated with infection status, and insufficient coordination between emergency and specialist services. Addressing these obstacles requires dedicated personnel and system-level adaptations, which we incorporated into our study design.*"

**• Lack of Specificity in Objectives: The introduction mentions the aim of the project but does not specify the specific objectives or hypotheses. The authors could more clearly define the specific objectives or hypotheses of the study.**

**Response:**
We thank the reviewer for this valuable observation. We agree that specifying the objectives more explicitly in the Introduction will provide clearer context for the study design and outcomes. We have revised the final paragraph of the Introduction to state the specific objectives of the study.

**Revised Introduction text (addition in *italic*):**
"In this study, we aimed to assess the results and impact of a healthcare quality improvement project toward providing HIV and HCV screening among patients seeking care in the ED of Hospital Clínic de Barcelona, in Spain. *Specifically, our objectives were: (1) to determine the uptake of HIV and HCV screening among eligible patients; (2) to estimate the prevalence of newly diagnosed infections; (3) to describe the demographic and clinical characteristics of patients with new infections; and (4) to provide LTC for both newly diagnosed and previously known but not retained patients.*"

**• Limited Context on Screening Protocols: The importance of screening is briefly mentioned, but the context on existing screening protocols and their limitations is not indicated. The authors could provide more background information on current HIV and HCV screening protocols, their limitations, and how the fast-track protocol addresses these issues, which would help readers better understand the significance of the study.**

**Response:**
We thank the reviewer for this important suggestion. We agree that providing more context on existing screening protocols and their limitations enhances the relevance and rationale for our fast-track model. Accordingly, we have revised the Introduction to add this background and clarify how our approach addresses identified gaps.

**Revised Introduction text (addition in *italic*):**
"In hospital settings, the European Centre for Disease Prevention and Control (ECDC) recommends that integrated testing for HIV/HCV be considered in patients who are diagnosed with any one of the three infections, belong to certain risk groups, have STI-compatible signs or symptoms, or who present with an HIV indicator condition [8]. In areas of high prevalence or incidence of STIs (and those with intermediate HCV prevalence or incidence), testing should be considered for any patient attending the emergency department (ED) or being admitted to hospital who undergoes a blood test for another indication and has never been previously tested for HIV/HCV infection [8]. *Despite these recommendations, screening in ED settings often remains ad hoc, dependent on individual clinical judgment, and inconsistently implemented. Common limitations include time constraints, lack of staff training, non-integrated workflows, and absence of standardized referral pathways for positive cases. Our fast-track protocol was specifically designed to overcome these barriers by embedding screening within routine workflows, automating eligibility and test ordering through electronic systems, and assigning dedicated staff to facilitate immediate linkage to care.*"

**2.1.3. Materials and Methods**

**• Study Design: The authors call this a retrospective observational study without specifying the specific type of observational study. This study would however fit better the definition of a retrospective cohort study because it involves identifying a cohort of patients based on their exposure status and then looking back at their medical records to assess outcomes related to HIV and HCV screening and care. I would suggest that the authors consider revising the type of study design accordingly.**

**Response:**
We thank the reviewer for this precise and constructive observation. We agree that the term “retrospective cohort study” more accurately reflects the design and methodology of our research. Accordingly, we have revised the description of the study design to reflect this clarification.

**Revised Materials and Methods text (addition in *italic*):**
"We conducted a retrospective *cohort ~~observational~~* study analyzing the results of this systematic opportunistic HIV and HCV screening program between 1st January 2020 and 31st December 2022."

**• Data Collection and Management: The methods for data collection and management are described, but there is no mention of how data quality and integrity were ensured. The authors could add more information on the procedures for ensuring data quality and integrity, such as validation checks, data cleaning processes, and handling missing data.**

**Response:**
We appreciate the reviewer’s suggestion and agree that including details on the data quality assurance process enhances methodological transparency. We have revised the *Materials and Methods* section to include a description of the procedures used to ensure data integrity and completeness during the analysis phase.

**Revised Materials and Methods text (addition in *italic*):**
"All data points were analyzed descriptively; the resulting statistics are reported as absolute values and/or percentages. *Data extraction was performed using predefined queries applied to the hospital's electronic health records system. Validation checks were conducted to ensure consistency across variables, and records were cross-referenced with laboratory and appointment databases to confirm test results and linkage to care status. Data cleaning included removal of duplicates, logical consistency verification (e.g., matching test results with timestamps and visit types), and manual review of records with missing or implausible values. Cases with missing data for key variables were excluded from the corresponding sub-analyses*."

**• Health Area covered by the study: Most of the information in this sub-section may not be appropriate to be placed in the section. It is suggested that the authors move the detailed description of the health area covered by the study to the background section to help readers understand the context and relevance of the study location. The authors can however retain the concise description of the area of study, sites, and source population that is directly related to the study methodology in the methods section.**

**Response:**
We thank the reviewer for this valuable recommendation. We agree that relocating the broader contextual information about the health area to the Introduction section improves the logical structure of the manuscript, while retaining only the essential methodological details in the Methods section. Accordingly, we have moved the relevant descriptive content from the *Health Area Covered by the Study* subsection to the Introduction, and revised the Methods section to maintain a concise reference to the setting.

**Revised Introduction text (addition in *italic*):**
"In this study, we aimed to assess the results and impact of a healthcare quality improvement project toward providing HIV and HCV screening among patients seeking care in the ED of Hospital Clínic de Barcelona, in Spain, *which is the primary healthcare provider for approximately 540,000 residents across several districts of the city, and a referral center for various medical specialties for patients from the broader Catalonia regions of Vallès Oriental and Osona. This context underscores the relevance and reach of implementing systematic screening interventions in such an urban tertiary care setting."*

**Revised Materials and Methods text (reduction to essential content):**
"In January 2020, we implemented a project to provide fast-track screening and immediate referral to specialist care in a novel STI outpatient clinic for adults in Hospital Clínic de Barcelona*, a tertiary university hospital serving a large urban population. The screening program was implemented in its emergency department and associated outpatient STI clinic."*

**• Statistical Analysis: The statistical analysis section is very brief and lacks detail on the specific statistical methods used. The authors should consider including details of the specific statistical tests and methods used, e.g. descriptive statistics (measures of central tendency and dispersion, and frequency distribution for categorical variables), chi-square tests, logistic regression or survival analysis. The authors could also specify the software and tools employed, e.g. SPSS (with version), R (with version) etc.**

**Response:**
We thank the reviewer for this helpful suggestion. While our study was based primarily on descriptive analyses, we agree that it is important to clarify the exact statistical methods and tools used. We have expanded the *Statistical Analysis* section to provide greater detail on the procedures employed, and included the software version used.

**Revised Materials and Methods – Statistical Analysis section (addition in *italic*):**
"All data points were analyzed descriptively; the resulting statistics are reported as absolute values and/or percentages. *To quantify the precision of the testing uptake and seroprevalence rates for HIV Ab, HCV Ab, and HCV RNA screenings, confidence intervals (CIs) were calculated for each of these measures. For comparative analysis, demographic groups were compared by Pearson’s Chi-squared test. P-values were estimated using Monte Carlo simulation with 10,000 replicates to account for small expected counts and ensure statistical robustness. All statistical analyses were performed using R version 4.3.3 (2024-02-29 ucrt).*"

**2.1.4. Results**

**• Lack of Statistical Detail: The results section lacks detailed statistical analysis, including p-values, other specific statistical tests, etc. I would recommend that the authors include detailed statistical results for each comparison, such as p-values, and specify the statistical tests used (e.g., chi-square test, t-test, etc).**

**Response:**
We thank the reviewer for this insightful comment. As our study primarily aimed to describe the implementation and outcomes of a quality improvement intervention, we initially focused on reporting descriptive statistics. However, we agree that further statistical analysis is needed. In response, we calculated the confidence interval (CI) of the testing uptake and seroprevalence of all our results from the HIV Ab, HCV Ab, and HCV RNA screening, and updated Table 1 and the results section accordingly.

**• Comparative Analysis: Comparative analysis is not provided between different groups (e.g., age groups, gender) for new infections and LTC rates. The authors should consider these comparative analyses between different demographic groups and provide statistical significance for these comparisons.**

**Response:**
We thank the reviewer for this important observation. We acknowledge that performing and reporting comparative analyses between demographic groups would enrich the interpretation of our findings. In response, we have updated Table 2 to include a Pearson's Chi-squared test, p-value was estimated by Monte Carlo simulation (10000 replicates).

**• Definition of acute HIV infection: The authors used positive p24 antigen to determine acute HIV infection. However, as the usual way to confirm acute HIV infection with p24 antigen is together with a negative or indeterminate HIV antibody test result, the authors should consider revising the manuscript accordingly.**

**Response:**
We thank the reviewer for this important clarification. We agree that the standard definition of acute HIV infection involves a positive p24 antigen result in combination with a negative or indeterminate HIV antibody result, reflecting the seroconversion window period. In our study, this more precise diagnostic criterion was not systematically recorded; therefore, we acknowledge the limitation and have revised the manuscript to reflect a more appropriate wording that avoids misclassification.

**Revised Results text (change in terminology – addition in *italic*):**
"Out of the 9,304 patients tested for HIV Ab, 38 new HIV infections were diagnosed (0.41% seroprevalence), *17 of which (45%) had a positive p24 antigen test result suggestive of acute or early-phase infection.*"

**• Patients re-linked to care: There is no context provided for the patients who were tested outside the screening program. It is also not clear why the authors reported on the indicators used. There is need for the authors to revise this section, providing context and presenting indicators that align better with the objectives of the study.**

**Response:**
We thank the reviewer for this insightful comment. We acknowledge that the inclusion of patients re-linked to care may appear disconnected from the study objectives if not adequately contextualized. These patients were not screened de novo within the project but were identified through routine HIV/HCV testing or hospital records during unrelated visits to the ED or STI clinic. As our quality improvement initiative also aimed to enhance retention in care, reporting these re-linkage outcomes was integral to demonstrating the program’s broader impact on public health. We have revised the *Results* and *Discussion* sections to clarify the source and relevance of these patients and aligned the indicators reported with the stated objectives.

**Revised Results text (addition in *italic*):**
"Besides the patients who were tested as part of this study, *individuals with known HIV or HCV infections who had not been engaged in care at the time of their visit were identified. These individuals were detected through routine serological testing or existing electronic health record alerts and were re-engaged via the same linkage mechanisms used in the screening protocol. A total of 298 HIV Ab-positive and 25 HCV Ab-positive patients were successfully re-linked to specialist care."*

**Revised Discussion text (addition in *italic*):**
*"Beyond detecting new infections, a key strength of this program was the ability to re-engage patients with previously known HIV or HCV diagnoses who had disengaged from care. This aligns with global health strategies that emphasize re-linkage as an essential element of the continuum of care, especially in high-throughput settings like EDs where such patients may sporadically access the healthcare system."*

**2.1.5. Discussion**

**• Lack of Detailed Comparative Analysis: Though the authors compared the findings with some previous studies, this is inadequate, and in most cases, little attempt to identify similarities and differences together with reasons for any differences. It is recommended that the authors address these issues.**

**Response:**
We appreciate the reviewer’s constructive feedback. We agree that enhancing the comparative analysis with previously published studies—including highlighting specific similarities, differences, and potential explanations—will strengthen the Discussion and improve interpretation of our findings. Accordingly, we have revised the *Discussion* section to expand the comparisons with similar emergency departments or urban screening settings.

**Revised Discussion text (additions in *italic*):**
"*Compared to other programs implemented in emergency departments (EDs), our detection rates fall within the low to intermediate range reported internationally. For instance, Simmons et al. conducted a systematic review on blood-borne virus screening in EDs, finding HIV prevalence rates ranging from 0.8% to 13%, and up to 17% for HCV (9). Most European studies included in that review reported rates closer to 1%, making our findings consistent with an urban setting with a moderate infection burden. In Portugal, Vaz-Pinto and colleagues implemented an automated HIV screening program in EDs and found a 0.55% prevalence, which is very similar to ours (14).*”

**• Context and interpretation of findings: There is inadequate context or interpretation of the findings. The authors could provide more context for the findings, explaining their relevance to public health and clinical practice and discussing the broader implications of the study results.**

**Response:**
We thank the reviewer for this valuable suggestion. We agree that more fully contextualizing our findings and explicitly outlining their implications for public health and clinical practice would enhance the Discussion. We have expanded the relevant paragraphs to discuss how our results contribute to the current understanding of integrated screening and LTC strategies, their relevance to WHO elimination goals, and their potential scalability in other settings.

**Revised Discussion text (addition in *italic*):**
"The results of our fast-track screening and referral program indeed show high linkage rates for new infections and considerable numbers of previously diagnosed patients being re-engaged with care services, which represent positive outcomes in light of the current recommendations and goals aimed at ending HIV and viral hepatitis epidemics. *These findings support the role of EDs and hospital-based STI clinics as critical touchpoints for identifying individuals who may not be reached through conventional screening in primary care settings. Our model also demonstrates that integrating LTC mechanisms into routine hospital workflows can achieve performance levels that meet or exceed global public health benchmarks.*

*In terms of broader implications, our study highlights that systematized, opportunistic BBV screening in urban ED settings is feasible and highly effective in identifying both new and previously known cases requiring care. This approach can serve as a replicable model for other healthcare systems, particularly in urban centers, willing to operationalize the WHO 2030 targets for HIV and HCV elimination. From a clinical standpoint, embedding these protocols helps reduce missed diagnostic opportunities and facilitates earlier engagement in care, ultimately improving long-term patient outcomes and reducing community transmission.*"

**2.1.6. Conclusion**

**• Lack of Specific Recommendations: The conclusion does not provide specific recommendations for practice or policy based on the study findings. The authors could include clear and actionable recommendations for healthcare providers and policymakers to improve HIV and HCV screening and linkage to care (LTC) in emergency department settings.**

**Response:**
We thank the reviewer for this important suggestion. We have revised the conclusion to include clear and actionable recommendations for healthcare providers and policymakers to enhance HIV and HCV screening and linkage to care in emergency departments.

**Revised Conclusion text (addition in *italic*):**
"Our study shows positive outcomes for a fast-track screening and LTC protocol for HIV and viral hepatitis. *~~The HIV and HCV prevalence rates found in our project suggest that adults seeking care in specialized EDs in urban settings should be considered for robust, opportunistic BBV screening.~~ Based on our findings, we recommend that healthcare institutions implement routine, opt-out HIV and HCV screening in emergency departments using automated testing algorithms and electronic medical record alerts. Additionally, the inclusion of dedicated staff responsible for real-time follow-up and linkage to care coordination is essential. Policymakers should prioritize funding and resource allocation to support such programs, which are critical for achieving national and global viral hepatitis and HIV elimination goals.* The high LTC rates achieved in this study indicate that immediate referral to specialist care is feasible in this setting using dedicated staff."

**• Absence of Future Research Directions: The directions for future research are not mentioned. The authors could highlight specific areas for future research.**

**Response:**
We thank the reviewer for this valuable suggestion. We have added a brief paragraph outlining potential directions for future research to the Discussion section.

**Revised Discussion text (addition in *italic*):**
*"Future research should focus on evaluating the long-term clinical outcomes of patients diagnosed through fast-track screening, including retention in care and treatment success rates. Additionally, studies exploring cost-effectiveness, patient acceptability, and the implementation of similar programs in diverse healthcare settings would further inform the scalability and sustainability of such interventions. Investigating barriers to screening uptake among subpopulations less likely to engage in testing could also enhance targeted strategies."*

**2.1.7. General comments: Language and Structure**

**• Though the manuscript is written predominantly in the third person, there are some areas where the first person is used. It is recommended that the authors revise the manuscript to ensure that it is consistently written in the third person.**

**Response:**
We thank the reviewer for this editorial observation. We have carefully reviewed the manuscript and revised all instances of first-person language to ensure consistent use of the third person throughout the text, in accordance with the journal’s stylistic conventions and scientific writing standards.

**• The terminology used in the manuscript is not always consistent. The authors should review the manuscript and ensure consistency in the terminology throughout the document.**

**Response:**

We thank the reviewer for highlighting this important point. We have thoroughly reviewed the manuscript and revised the terminology to ensure consistent use of key terms throughout the text.

**• The manuscript generally does not flow well, and it has a number of grammatical errors. Some sentences are also long and winding, and thus unclear, for example lines 68-72, lines 91—95 and paragraph with lines 199-206. The authors will need to proofread all sections of the manuscript and revise it to improve the language, structure, clarity and general flow.**

**Response:**
We thank the reviewer for this detailed and helpful comment. In response, we have thoroughly revised the manuscript for grammar, clarity, and flow. Long and complex sentences were simplified and restructured to enhance readability.

**2.2. Minor Issues**

**2.2.1. Abstract**

**• HIV and HCV tests used were not detailed: The abstract mentions that HIV and HCV screening were conducted but does not specify the tests used. It is recommended that the authors mention the tests carried out to provide readers with a clearer understanding of the study methodology.**

**Response:**
We thank the reviewer for this helpful suggestion. To improve clarity and methodological transparency, we have added a brief mention of the specific tests used for HIV and HCV screening in the Abstract.

**Revised Abstract text (addition in *italic*):**
"Using the FOCUS TEST model as a framework, we conducted systematic opportunistic HIV and HCV screening and offered linkage to care (LTC) to patients with positive test results. *Screening was performed using fourth-generation chemiluminescence immunoassays for HIV and HCV antibodies, including p24 antigen detection, with confirmatory HCV RNA testing by PCR.*"

**2.2.2. Introduction**

**• Insufficient Justification for Study Setting: The choice of Hospital Clínic de Barcelona as the study setting is mentioned but not justified. It is suggested that the authors explain why this particular hospital was chosen for the study, the patient demographics, previous screening efforts and relevance to the study objectives.**

**Response:**
We thank the reviewer for this useful suggestion. We agree that providing a brief justification for selecting Hospital Clínic de Barcelona as the study site would enhance the contextual understanding of our project. We have therefore expanded the Introduction to include relevant information on the hospital.

**Revised Introduction text (addition in *italic*):**
"In this study, we aimed to assess the results and impact of a healthcare quality improvement project toward providing HIV and HCV screening among patients seeking care in the ED of Hospital Clínic de Barcelona, in Spain, which *is the primary healthcare provider for approximately 540,000 residents across several districts of the city, and a referral center for various medical specialties for patients from the broader Catalonia regions of Vallès Oriental and Osona. This context underscores the relevance and reach of implementing systematic screening interventions in such an urban tertiary care setting."*

**2.2.3. Materials and Methods**

**• The FOCUS TEST Model: The description of the FOCUS TEST model is informative but does not show how the pillars specifically contributed to the study. The authors could expand the description to include examples of how each pillar was implemented in the study.**

**Response:**
We thank the reviewer for this constructive suggestion. We agree that providing concrete examples of how each pillar of the FOCUS TEST model was operationalized in our project would clarify its practical application and value. We have revised the description in the *Materials and Methods* section accordingly.

**Revised Materials and Methods text:**
"We applied the FOCUS TEST model to encourage systems change and to extend the adoption of screening and LTC [18]. “TEST” stands for four pillars:

- *Testing*: Screening was embedded into routine ED workflows for eligible patients, using existing clinical staff and sample collection protocols.
- *Electronic health record (EHR) enhancements*: The EHR system was modified to include automated prompts and predefined order sets that facilitated rapid test ordering and eligibility checks.
- *Systemic policy change*: An institutional protocol was adopted mandating HIV and HCV screening for all patients meeting risk criteria, ensuring consistency across shifts and personnel.
- *Training and feedback*: Regular training sessions were conducted for ED clinicians and nurses, accompanied by monthly feedback reports on screening volume, positivity rates, and LTC performance, which helped maintain program engagement and quality.*"*

**• Immunoassay Tests for Screening: The authors mention the immunoassays that they used but did not provide specific details on these immunoassays. As it is generally preferred in published literature to provide specific details about the immunoassay tests used as this enhances transparency, reproducibility, and allows for better comparison across studies, it is suggested that the authors provide the names of the assays and their manufacturers.**

**Response:**
We appreciate the reviewer’s comment and agree that providing the specific names and manufacturers of the immunoassay tests enhances methodological transparency and reproducibility. We have revised the *Materials and Methods* section to include this information.

**Revised Materials and Methods text (addition in italic):**
"We used fourth-generation chemiluminescence immunoassays to determine antibodies against HIV and HCV, as well as p24 antigen. *Specifically,* *HIV Ag/Ab Combo (Siemens, Sistema Atellica) and HCV (Siemens, Sistema Atellica).* HCV viral load determination was calculated through real-time PCR on *the automated system COBAS® AmpliPrep/COBAS® TaqMan® HCV, v2.0 (Roche, Sistema COBAS)*.”

**• Variables and Outcomes: The variables and outcomes are listed, but the rationale for selecting these specific variables is not discussed. The authors could provide a justification for the selection of these variables, explaining their relevance to the study objectives.**

**Response:**
We thank the reviewer for this useful observation. We agree that explaining the rationale for selecting the reported variables and outcomes strengthens the methodological transparency and supports their alignment with the study objectives. Accordingly, we have revised the *Materials and Methods* section to include this clarification.

**Revised Materials and Methods text (addition in *italic*):
*"***Variables considered in this study were: age (categorized in five strata: 18–22, 23–30, 31–40, 41–50, 51–90), sex, and injected drug use.
For HIV and HCV, the main outcomes reported in this analysis were: number of tests performed, testing uptake (defined as the percentage of patients undergoing testing out of the total number of patients who were offered it), number of new infections (i.e., tests with a positive result), seroprevalence (defined as the percentage of patients with a positive test result out of the total number of patients who underwent testing), and rate of LTC (defined as the percentage of patients attending a first medical appointment after diagnosis out of the total number of patients with a positive test result).
*These variables were selected based on their clinical and public health relevance and their alignment with international screening and LTC indicators. Demographic factors such as age and sex are known to influence infection risk and care engagement. Uptake, prevalence, and LTC are critical for assessing the effectiveness and operational feasibility of a screening program, and are key metrics in evaluating progress toward WHO elimination targets."*

**2.2.4. Results**

**• Presentation of Data: The presentation of data in the text is not well structured and is not easy to follow. It is recommended that the authors improve description and presentation of the data in the text.**

**Response:**
We thank the reviewer for this important feedback. We have revised the *Results* section to improve readability and comprehension.

**• Presentation of data in Table 2: The text description of the data in Table 2 is not clear, and the authors could revise the text to ensure a more complete description of the results and improved flow.**

**Response:**
We thank the reviewer for this helpful suggestion. We have revised the narrative accompanying Table 2 to ensure a more complete, structured, and comprehensible description of the results.

**2.2.5. Discussion**

**• Clarity and Structure: The discussion section is not well structured and does not flow well. It is recommended that the authors revise the discussion section to ensure that it flows better and improves clarity.**

**Response:**
We thank the reviewer for this valuable recommendation. In response, we have revised the *Discussion* section to improve clarity, coherence, and logical progression.

**• Insufficient Explanation of Limitations: Though the limitations of the study are enumerated, they are not discussed in sufficient detail. The authors could expand on the limitations, providing a more comprehensive explanation of how they might affect the study results and interpretations. Potential biases and confounding factors could also be discussed in greater detail.**

**Response:**
We thank the reviewer for this important comment. We agree that a more in-depth discussion of the study’s limitations, including potential sources of bias and confounding, is warranted. Accordingly, we have expanded the relevant paragraph in the *Discussion* section to provide a more comprehensive explanation of how these limitations may influence the interpretation and generalizability of our findings.

**Revised Discussion text (addition in *italic*):**
"This study has some limitations that should be considered to appropriately interpret the findings. First, data were collected at a single site in Barcelona, which may limit the generalizability of our findings to other regions with different sociodemographic characteristics or different epidemiology of HIV and HCV infections. *This may be particularly relevant in rural or non-European contexts where emergency department utilization patterns and access to STI/HIV services differ significantly.*

Second, due to the study setting and eligibility criteria, the population analyzed consisted of patients presenting with high-risk factors or exposed to high-risk behaviors. This could have introduced selection bias to some extent, and therefore our findings may not necessarily be representative of the general population (as previously discussed). *Additionally, individuals who declined testing may differ systematically from those who accepted, potentially affecting the observed seroprevalence and LTC rates.*"

**2.2.6. Conclusion**

**• Insufficient Summary of Key Findings: The conclusion does not adequately summarize the key study findings. The authors could provide a concise summary of the main results.**

**Response:**
We thank the reviewer for this helpful suggestion. We agree that a clearer and more concise summary of the key findings would strengthen the conclusion and reinforce the study’s contribution.

**________________________________________**
